# A Remarkable Impact of pH on the Thermo-Responsive Properties of Alginate-Based Composite Hydrogels Incorporating P2VP-PEO Micellar Nanoparticles

**DOI:** 10.3390/polym16070886

**Published:** 2024-03-24

**Authors:** Amalia Iliopoulou, Zacharoula Iatridi, Constantinos Tsitsilianis

**Affiliations:** Department of Chemical Engineering, University of Patras, 26500 Patras, Greece; up1066385@upnet.gr (A.I.); iatridi@upatras.gr (Z.I.)

**Keywords:** heterograft copolymer, hydrogel, alginate, thermo-responsive, pH-responsive, poly(2-vinyl pyridine)-*b*-polyethylene oxide, diblock copolymer micelles, drug delivery

## Abstract

A heterograft copolymer with an alginate backbone, hetero-grafted by polymer pendant chains displaying different lower critical solution temperatures (LCSTs), combined with a pH-responsive poly(2-vinyl pyridine)-*b*-poly(ethylene oxide) (P2VP-*b*-PEO) diblock copolymer forming micellar nanoparticles, was investigated in aqueous media at various pHs. Due to its thermo-responsive side chains, the copolymer forms hydrogels with a thermo-induced sol–gel transition, above a critical temperature, T_gel_ (thermo-thickening). However, by lowering the pH of the medium in an acidic regime, a remarkable increase in the elasticity of the formulation was observed. This effect was more pronounced in low temperatures (below T_gel_), suggesting secondary physical crosslinking, which induces significant changes in the hydrogel thermo-responsiveness, transforming the sol–gel transition to soft gel–strong gel. Moreover, the onset of thermo-thickening shifted to lower temperatures followed by the broadening of the transition zone, implying intermolecular interactions between the uncharged alginate backbone with the PNIPAM side chains, likely through H-bonding. The shear-thinning behavior of the soft gel in low temperatures provides injectability, which allows potential applications for 3D printing. Furthermore, the heterograft copolymer/nanoparticles composite hydrogel, encapsulating a model hydrophobic drug in the hydrophobic cores of the nanoparticles, was evaluated as a pH-responsive drug delivery system. The presented tunable drug delivery system might be useful for biomedical potential applications.

## 1. Introduction

Hydrogels are three-dimensional (3D) network structures formed through chemical or physical cross-links or chain entanglements. Stimuli-responsive hydrogels, also known as “smart” hydrogels, are materials that respond to various stimuli (pH, temperature, light, ionic strength, magnetic, redox, electric, and chemical response), exhibiting reversible changes in their chemical and physical state [1,2,3,4]. Due to their soft texture, elasticity, biocompatibility, high swelling capability, and similarity to body tissues, hydrogels are ideal candidates for use as biomaterials in a variety of applications [5,6] like delivery of drugs and other bioactive ingredients [7,8,9,10,11,12,13,14,15,16], 3D printing [17,18,19,20], tissue engineering, repair and regeneration [21,22,23,24], or shear-thinning injectable matrices for bioapplications [25,26].

Nowadays, various stimuli-responsive hydrogels, originating from synthetic as well as natural resources, are extensively used as biomaterials in several bioapplications, including tissue engineering, drug delivery, etc. Mostly known examples of synthetic hydrogels as biomaterials include polymeric and composite systems. On the other hand, common biomaterials of natural origin are polysaccharide-based or protein-based biomaterials. Polysaccharides are non-synthetic polymers that can be isolated from a variety of natural sources like plants, animals, or algae. Some examples of polysaccharides are chitosan, hyaluronan, pullulan, dextran, xanthan, cellulose, pectin, guar gum, collagen, gelatin, agar, alginate, carrageenan, and silk. This class of materials has gained scientific interest over synthetic polymers due to their exceptional advantages such as biocompatibility, biodegradability, hydrophilicity, environmentally friendly, and low or non-toxicity.

The Incorporation of thermo-sensitive groups in polysaccharides creates new “smart” materials with thermo-responsive behavior [27,28,29]. Interestingly, such materials can present thermo-induced sol-to-gel transition properties, rendering them ideal candidates for use as potential injectable hydrogels [30]. In this respect, thermo-sensitive polymers like poly(N–isopropylacrylamide) (PNIPAM) are grafted onto or cross-linked with polysaccharide backbones. PNIPAM is one of the most extensively studied thermo-responsive polymers exhibiting a lower critical solution temperature (LCST) of around 32 °C, close to physiological body temperature. Its polymer chains are hydrophilic and water soluble at temperatures below the LCST, but above the LCST, they become hydrophobic and their solubility in water is not favorable anymore, leading to phase separation [31]. The LCST behavior of PNIPAM can be finely altered and tuned to desired values simply by the addition of hydrophobic or hydrophilic monomers. The addition of more hydrophobic monomers like N-tert-butyl acrylamide (NtBAM) decreases the LCST [32,33], while the incorporation of more hydrophilic monomers such as acrylic acid shifts LCST to higher temperatures [34]. Many interesting articles report the thermo-induced sol-to-gel transition of alginate polysaccharide grafted with PNIPAM chains [35,36,37,38,39,40]. Our group has long experience in the design and study of thermo-responsive graft copolymers comprising an alginate backbone and thermo-sensitive NIPAM-based homopolymers or copolymers [32,41,42]. It has been shown that such polymers, due to shear-thinning and thermo-thickening effects, can form hydrogels with injectability and self-healing capabilities, rendering them ideal applicants for use in fields like tissue engineering and therapeutic cargo delivery.

The incorporation of organic or inorganic particles into polymeric scaffolds like hydrogels has been proven to better control the pharmacokinetic profiles of drug-loaded molecules as well as to improve their stability and increase local drug concentration, providing a drug reservoir at the site of administration [43,44,45,46,47,48,49]. Moreover, it has been reported that hydrogel/nanoparticle complex systems display improved rheological and mechanical properties, which in turn enhance their printability and injectability [38,50,51].

**Scheme 1:**
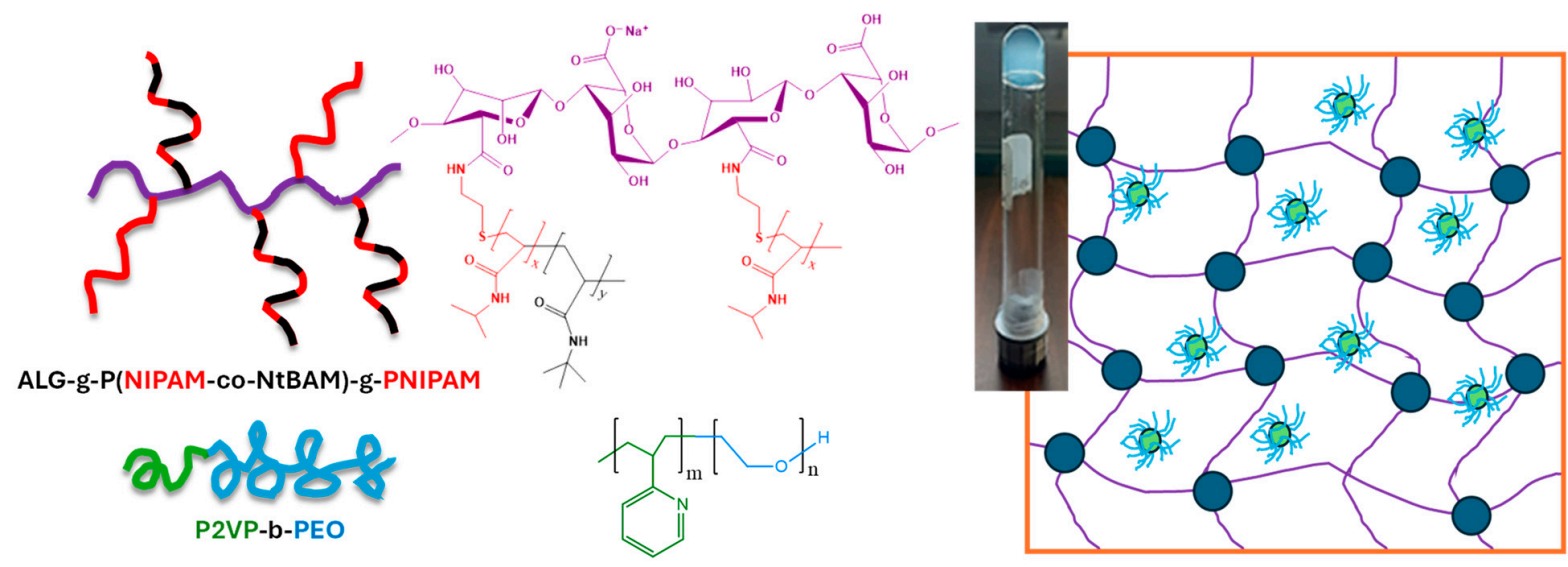
Schematic representation of the composite hydrogel. The digital photo demonstrates a free-standing gel prepared at room temperature and pH 3.5.

In this paper, we report on a composite hydrogel constituted of an alginate heterografted gelator, exhibiting thermo-thickening behavior through a sol–gel transition at T_gel_ of 34.5 °C, encapsulating polymeric nanoparticles, formed by the spontaneous self-assembly of P2VP-*b*-PEO diblock copolymers (Scheme 1). Thanks to the pH-dependent properties of P2VP (cationic/hydrophobic), the rheological behavior of the composite was focused on a pH range of 3.5–5.4 in the vicinity of its pK_a_. The data showed a remarkable influence of pH on the thermo-induced gelation of the alginate copolymer, namely, elasticity enhancement, shift of the critical thermo-thickening temperature at lower values, and broadening of the gelation transition zone. Surprisingly, the same behavior was observed for the gelator without nanoparticles, showing for the first time that pH is another stimulus that significantly affects the thermo-responsiveness of this kind of PNIPAM-based alginate graft copolymers. These results are attributed to secondary intermolecular interactions between the protonated repeating units of the alginate backbone and the PNIPAM side chains. Importantly, the hydrogel can be formed at a pH below 4.0, even at room temperature without the necessity of heating, which is beneficial for 3D-printing stabilization. Finally, the composite was evaluated as a pH-dependent drug delivery system using Nile Red as a model hydrophobic drug. For this purpose, a pH-dependent hydrophobic core of diblock copolymer micellar nanocarriers was used to encapsulate the drug.

## 2. Materials and Methods

### 2.1. Materials

The synthesis of the heterograft copolymer ALG-g-P(NIPAM_86_-co-NtBAM_14_)-g-PNIPAM (denoted by ALG-g-HG) has been previously described [42]. A linear diblock P2VP-*b*-PEO copolymer was created in our lab by anionic “living” polymerization and sequential addition of monomers, according to the literature [52]. The molecular characteristics of ALG-g-HG and P2VP-*b*-PEO copolymers are presented in Tables S1 and S2, respectively (see Supplementary Materials). Nile Red was purchased from Sigma-Aldrich (St. Louis, MO, USA). Standard solutions of hydrochloric acid (HCl) and sodium hydroxide (NaOH) were purchased from Panreac (Chicago, IL, USA) and used as received, without further purification. Disodium hydrogen phosphate (Na_2_HPO_4_) and potassium dihydrogen phosphate (KH_2_PO_4_) were obtained from Carlo Erba (Milan, Italy) and Merck (Darmstadt, Germany), respectively, and used as received for the preparation of two PB buffer solutions (10 mM, pH 3.5 or pH 7.4). The organic solvent tetrahydrofuran (THF) was obtained from Sigma-Aldrich (St. Louis, MO, USA). An ELGA Medica-R7/15 device (ELGA Labwater, Woodridge, IL, USA) was used to produce ultrapure water.

### 2.2. Preparation of Aqueous Polymer Solutions and Rheology Study

Aqueous solutions of ALG-g-HG copolymer or hybrid ALG-g-HG/P2VP-*b*-PEO systems were prepared and left under stirring at room temperature until homogeneity. In all solutions, the concentration of heterograft ALG-g-HG copolymer was set at 4 wt%, while the concentration of the P2VP-*b*-PEO micelles in the ALG-g-HG/P2VP-*b*-PEO hybrids was set at 4 wt%. The pH of the solutions was set at the desired values, using HCl (1 M) or NaOH (1 M). The rheological study of the ALG-g-HG heterograft copolymer and hybrid ALG-g-HG/P2VP-*b*-PEO system was performed using an AR-2000ex stress-controlled rheometer (TA Instruments, New Castle, DE, USA) equipped with a Peltier system for controlling the temperature and a solvent trap to avoid concentration changes owing to water evaporation. Cone-plate geometry was used (diameter 20 mm, angle 3°, truncation 111 μm), and the experiments were performed in the linear viscoelastic regime (LVR), which was determined by strain sweep tests at a frequency of 1 Hz.

### 2.3. Loading and Release of Nile Red from P2VP-b-PEO Micelles

The P2VP-*b*-PEO micelles were prepared by the film hydration method. In brief, predetermined amounts of P2VP-*b*-PEO copolymer and the target model drug (Nile Red) were solubilized in THF. The theoretical (feed) loading (Lth %) in the polymeric micelles was equal to 9.98%, as calculated by Equation (1):

(1) $$Lth\;\%=\frac{w_{0}}{w_{m}+w_{0}}\times \;\;100$$

where *w*_0_ and *w_m_* are the amounts (μg) of the initially added probe (Nile Red) and amount of polymer micelles, respectively.

At the next step, THF was removed using a rotary evaporator until a thin polymer/Nile Red film was formed. Afterwards, ultrapure water was added to solubilize the film and form the P2VP-*b*-PEO copolymer micelles, which were loaded with Nile Red (P2VP-*b*-PEO@Nile Red). Finally, the Nile Red loaded micelles were obtained in a solid state using freeze drying. In order to determine the encapsulation efficiency (EE %) and the loading efficiency (LE %) of Nile Red in the P2VP-*b*-PEO copolymer micelles, two calibration curves were constructed in THF/PB buffer at pH 3.5 and THF/PB buffer at 7.4, where the volume ratio of THF to PB buffers was 60/40 *v*/*v*.

For these calibration curves, different concentrations of Nile Red (ranging from 0.5 to 15 μg/mL) in THF/PB buffer solutions were prepared and their absorbance values were recorded on a Hitachi U–2001 UV–VIS spectrophotometer (Schaumburg, IL, USA). Absorbances were determined at the lambda maximum (550 nm), and probe concentrations were quantified using molar absorption coefficients (ε) determined in each THF/PB buffered solution (pH 3.5; pH 7.4).

The loading efficiency (LE %) and encapsulation efficiency (EE %) of Nile Red were calculated using Equations (2) and (3), respectively:

(2) $$LE\;\%=\frac{w}{w_{m}+w}\;\times \;100$$

(3) $$EE\;\%=\frac{w}{w_{0}}\;\times \;100$$

where *w*, *w*_0_, and *w_m_* are the amounts (μg) of an entrapped model drug, initially added probe (Nile Red), and amount of polymer micelles, respectively.

As for the drug release kinetics study, a given amount (~5 mg) of lyophilized model drug-loaded micellar powder (P2VP–*b*–PEO@Nile Red) was dispersed in 1 mL of PB buffer with pH 3.5 (stimulating the stomach pH) or 7.4 (which is specific for blood and colonic fluids) and then these solutions were transferred into dialysis membranes (12 kDa MWCO). These membranes were immersed in 5 mL PB buffer solutions of pH 3.5 or pH 7.4, in glass bottles, and kept under stirring (100 rpm) in a water bath at 37 °C, in the dark, to avoid photo-degradation of the model drug. After 120 h of experiment, the dialysis membranes in 5 mL PB buffer solutions were subjected to sonication using an ultrasonic bath sonicator. All 5 mL PB samples were extracted at definite time intervals and replaced with newly added 5 mL of fresh PB solution in the glass bottles with the membranes. The extracted 5 mL samples were mixed with 7.5 mL of THF to achieve a mixture of THF/PB with a 60/40 ratio in volume and then used to spectrophotometrically quantify the amount of the released Nile Red at a wavelength of 550 nm. The amount of Nile Red from the micelles was spectrophotometrically quantified based on their calibration curve, as previously designed.

### 2.4. Release of Nile Red from ALG-g-HG/P2VP-b-PEO Hydrogel Composite System

Two 4 wt% ALG-g-HG aqueous solutions with pH adjusted to 3.5 and 7.4 were prepared, as described in Section 2.2; 12 mg of Nile Red loaded P2VP–*b*–PEO micelles (P2VP–*b*–PEO@Nile Red) were added to 1.2 mL of the prepared ALG-g-HG aqueous solutions. The mixtures were transferred in two dialysis membranes (MWCO 12 kDa), which in turn were immediately submerged in 5 mL of phosphate buffer, 10 mM of pH = 3.5 or 7.4, at 37 °C and shaken at 100 rpm in the dark. At specific timed intervals, the 5 mL of buffer was removed and renewed with 5 mL of fresh buffer. The cumulative release rate (%) of Nile Red was calculated in a similar way, as described in Section 2.3.

### 2.5. Techniques

Zeta potential and dynamic light scattering (DLS) measurements of ALG-g-HG and P2VP-*b*-PEO aqueous solutions were obtained from a Malvern Nano Zetasizer analyzer (Malvern, UK) equipped with a He–Ne laser at 633 nm. The final polymer concentration of ALG-g-HG was set at 0.4 %wt while the concentration of P2VP-*b*-PEO was set at 0.1 %wt (keeping a 4:1 ratio of ALG-g-HG:P2VP-*b*-PEO). The desired pH values of the solutions were achieved by adding HCl (1 M) or NaOH (1 M).

## 3. Results and Discussion

### 3.1. Rheological Investigation at Various pHs

**Figure 1:**
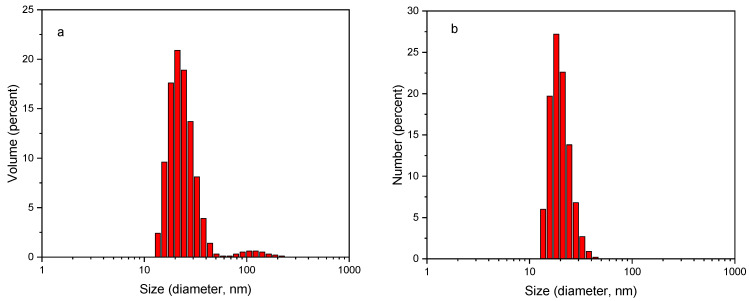
(**a**) Volume and (**b**) number average particle size distributions of 0.075 wt% P2VP-*b*-PEO aqueous solution at pH 7.4 and at 25 °C.

A hydrogel nanocomposite was designed, comprising a thermo-responsive alginate-based gelator, encapsulating polymeric nanoparticles (NPs) as nano-carriers. Particularly, the gelator used is the heterograft copolymer constituted of an alginate backbone, which is grafted by two different types of thermo-responsive polymer chains (ALG-g-HG): (a) pure poly(N–isopropylacrylamide) (PNIPAM) homopolymer and (b) poly[(N–isopropylacrylamide)–*co*–(N–tert–butyl acrylamide)] (P(NIPAM–*co*–NtBAM)) random copolymer, displaying different lower critical solution temperatures (LCST) [42]. The aqueous dispersion of 4 wt% ALG-g-HG, at pH 7.4, exhibits a sol–gel transition upon heating at T_gel_ = 34.5 °C as determined by oscillatory shear experiments at the G’/G” crossover point (Figure S1a). The NPs formed by spontaneous self-assembly of 1 wt% P2VP-*b*-PEO block copolymers in aqueous media of pH 7.4, forming star-like micelles of a core-shell structure with P2VP hydrophobic cores and PEO water-soluble shells. Note that P2VP is a pH-dependent cationic polyelectrolyte, exhibiting pK_a_ at pH 5.0, above which it becomes hydrophobic, driving hydrophobic association [53,54,55]. The hydrodynamic diameter of NPs at pH 7.4 was determined by dynamic light scattering at 23.56 nm (volume average, PDI 0.267) (Figure 1). Some bigger aggregates appearing in the volume-weighted size distribution are negligible in number as they disappear in the number-weighted distribution.

**Figure 2:**
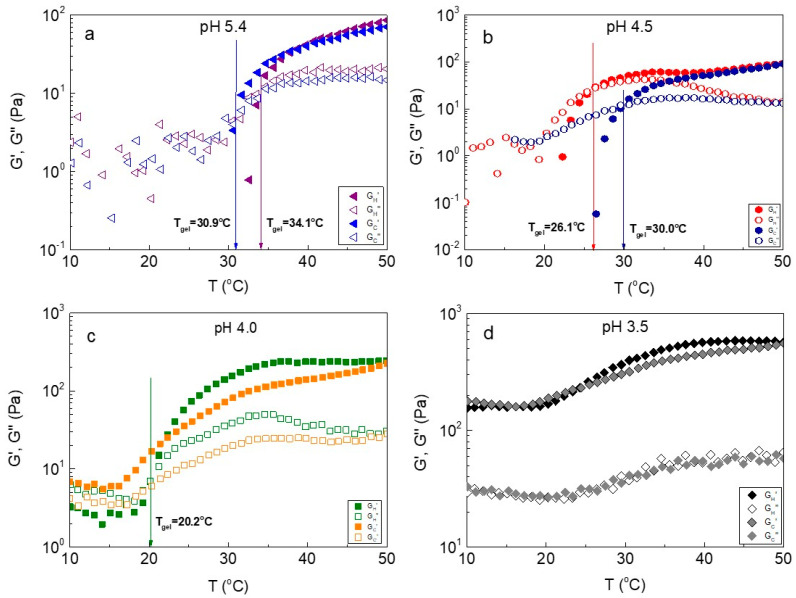
Storage (G’) and loss (G”) modulus as a function of the temperature of the ALG-g-HG/P2VP-*b*-PEO nanocomposite hydrogels at (**a**) pH 5.4, (**b**) pH 4.5, (**c**) pH 4.0, and (**d**) pH 3.5.

The final mixed aqueous solution under investigation contained 4 wt% ALG-g-HG and 1 wt% P2VP-*b*-PEO-based NPs. Nanocomposite hydrogels regulated at different pH were explored by oscillatory shear measurements to evaluate the potential influence of pH on their thermal and mechanical response. The pH window was chosen in the narrow range of 3.5–5.5, which lies in the vicinity of pK_a_ of P2VP in which its degree of ionization increases by lowering the pH (see z-potential versus pH in Supplementary Materials, Figure S2). Figure 2 demonstrates the storage (G’) and loss (G”) modulus as a function of temperature at different pH values. The experiments were conducted in the linear viscoelastic regime by applying a successive heating/cooling ramp with a rate of 1 °C/min. In all cases, the moduli exhibit strong temperature dependence revealing a thermo-thickening behavior that depends on the medium pH. At pH 5.4, a thermo-induced sol–gel transition can be observed, providing that the storage modulus increases sharply upon heating, surpassing the loss of one at the critical T_gel_ determined at 34.1 °C. The phenomenon is reversible as revealed by the cooling procedure, with slight hysteresis. The gel–sol transition was shifted at 30.9 °C, which is a few degrees lower than that observed by heating, due to kinetic effects [29]. This thermo-thickening behavior is quite similar to the formulation of 4 wt% ALG-g-HG without NPs (Figure S1b). This implies that the presence of P2VP-*b*-PEO-based micelles at pH 5.4 does not affect the network structure formed by the physical crosslinking of the thermo-responsive side chains of the gelator. However, by lowering the pH, remarkable changes in the characteristics of the hydrogel thermo-responsiveness can be observed. The main variation observed in the general thermal behavior of the formulations is that the sol–gel transition observed at pH 5.4 was transformed to a soft gel–strong gel transition at pH 3.5 since the G’/G” crosslinking disappeared and G’ predominates G” in the entire temperature range explored. At the intermediate pH 4.5 and 4.0, the thermo-responsive behavior exhibits additional significant hysteresis effects. Particularly, at pH 4.5, the transition point T_gel_ in the heating procedure was shifted to lower values of about 8 °C, which is notably more pronounced than in the cooling procedure. While at pH 4.0, T_gel_ (heating) dropped to 20.2 °C, which is about 14 °C lower than that at pH 5.4. Moreover, in the cooling procedure, the gel–sol transition did not occur and provided that the moduli, although it decreased upon lowering the temperature, never crossed each other, hence preserving the gel-like behavior at low temperatures.

**Figure 3:**
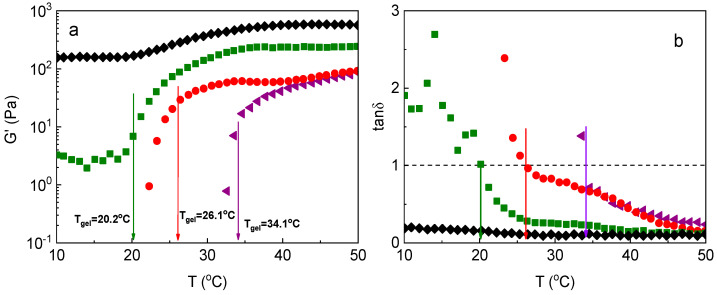
(**a**) Storage (G’) modulus and (**b**) tanδ as a function of the temperature of the ALG-g-HG/P2VP-*b*-PEO nanocomposite hydrogels at various pHs: pH 3.5 (black symbols); pH 4.0 (green symbols); pH 4.5 (red symbols); pH 5.4 (purple symbols).

We now focus on the data dealing with the heating process, which is related to potential biomedical applications. Figure 3 presents the temperature dependence of the elastic part of the complex modulus, G’ (Figure 3a) together with the loss tangent (G”/G’) at various pHs. By lowering the pH, two main effects can be observed: (a) a decrease of the sol–gel transition (defined at tanδ = 1, Figure 3b) and (b) the elasticity of the formulation is expanded at lower temperatures and simultaneously increases. Provided that G’ reflects the degree of crosslinking, this behavior should be ascribed to additional crosslinking leading to the mechanical reinforcement of the hydrogel. This is also reflected in tanδ, which decreases with the pH alongside the temperature variation.

**Figure 4:**
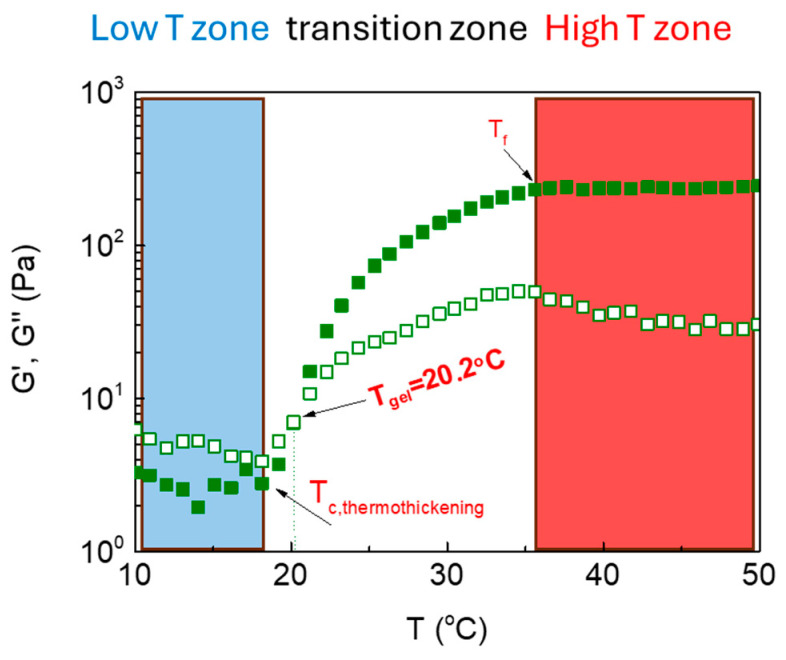
The three temperature zones designated in the storage (G’) and loss (G”) modulus versus the temperature plot of an ALG-g-HG/P2VP-*b*-PEO nanocomposite hydrogel.

To better qualitatively analyze the data, we can distinguish three temperature zones as indicated in Figure 4: the low-temperature zone, the high-temperature zone, and between them, the transition zone. The latter zone is defined as the region between the temperature at the onset of the abrupt increase of the elastic modulus G’, namely, T_c,thermo-thickening_ and T_f_, i.e., the temperature where G’ becomes independent of T or increases slightly and smoothly. A number of characteristic factors related to the thermo-thickening phenomenon based on the elastic modulus G’, i.e., T_c,thermo-thickening_, T_f_, T_gel_, ΔT (transition zone), and the extent of the thermo-thickening effect exemplified by the ratio of G’ augmentation (G’(T = 50)/G’(T_c,thermo-thickening_)) are gathered in Table 1 at various pHs.

**Figure 5:**
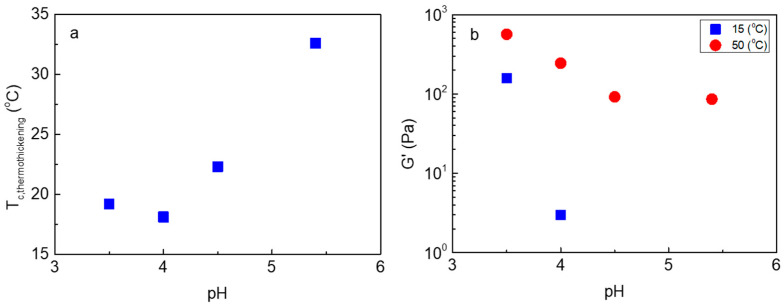
pH dependence of (**a**) T_c,thermo-thickening_ and (**b**) storage modulus (G’) at 15 and 50 °C, as obtained from Figure 3a, for the ALG-g-HG/P2VP-*b*-PEO nanocomposite hydrogels.

One of the important effects of pH concerns the transition zone. As indicated in Figure 5a, the onset of thermo-thickening is shifted to a lower temperature of more than 13 °C, from pH 5.4 to 3.5. This causes the broadening of the transition zone ΔΤ from 9.4 to 19.5 degrees and the shifting of the sol–gel transition to lower temperatures. As is known, the thermo-thickening effect is due to the thermal-induced hydrophobic interactions of the PNIPAM-based sticky side chains of the gelator, which are activated above their LCST, leading to an intermolecular association. The fact that this association starts occurring at lower temperatures implies that the solubility of the side chains was obstructed by other interactions. Indeed, focusing on the low-temperature zone, we observe a remarkable increase of G’ by more than two orders of magnitude (Figure 3a and Figure 5b), which leads to gel-like behavior even at low temperatures below T_gel_ at pH 3.5. This suggests the formation of a network different than that induced by heating, which is formed from the interactions, responsible for the variations (T_c,thermo-thickening_, T_gel_, ΔT) observed in the transition zone. Moreover, these new interactions seem to contribute as well to the G’ augmentation in the high-temperature zone (Figure 5b). Provided that the G’ augmentation is more pronounced in low temperatures, it eventually significantly weakens the extent of the thermo-thickening effect, as reflected by the G’(T = 50)/G’(T_c,thermo-thickening_) ratio, from 110.5 to 3.5 (Table 1). This likely suggests that a smaller number of side chains are now available for hydrophobic association.

**Figure 6:**
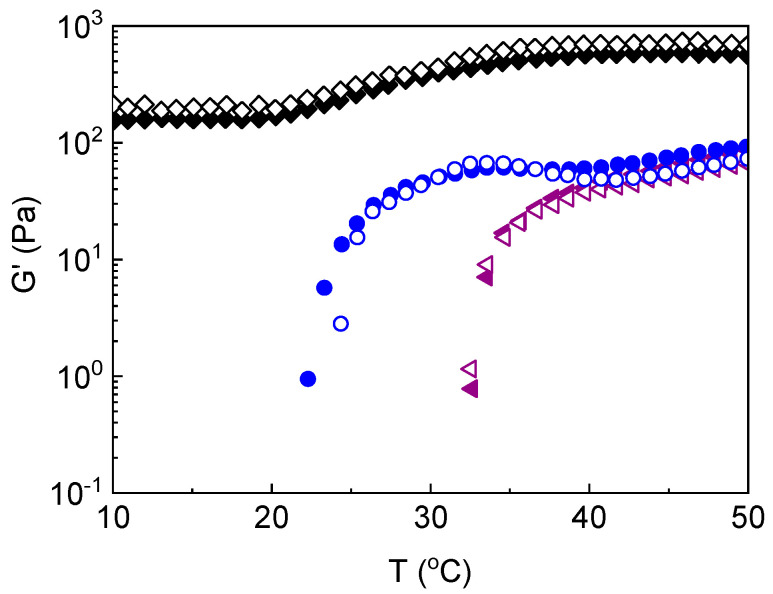
Storage modulus (G’) as a function of the temperature of the pure ALG-g-HG hydrogels (open symbols) and ALG-g-HG/P2VP-*b*-PEO nanocomposite hydrogels (solid symbols), at various pHs: pH 3.5 (black symbols); pH 4.5 (blue symbols); pH 5.4 (purple symbols).

In order to elucidate the nature of the interactions, other than the hydrophobic one induced by heating, that contributes to the reinforcement of the network, we also explored the behavior of the ALG-g-HG without the presence of NPs. Figure S3 demonstrates the data from the temperature ramp experiments performed in the linear viscoelastic regime (strain amplitude 0.1%) at 1 Hz of 4 wt% ALG-g-HG at pH 4.5 and 3.5, keeping the same conditions with the composite formulations. For the sake of comparison, these data have been plotted together with the data of Figure 3a and Figure S1b in Figure 6. Surprisingly, the behavior of the bare ALG-g-HG gelator is quite like those of the formulations incorporating the P2VP-*b*-PEO micellar NPs, since the data for the corresponding formulations at the same pH almost coincide. This suggests that the additional crosslinking of the ALG-g-HG, observed at low temperatures in the absence of PNIPAM hydrophobic association, must be attributed to the intermolecular interaction between the alginate backbone and the PNIPAM-based side chains. This is corroborated by the shift of the characteristics of the transition zone (T_c,thermo-thickening_, T_gel_) to the lower temperatures discussed above. Note that a gel-like behavior was not observed in pure alginate formulations of 4 wt% at the same pH range. At low pH, although the degree of ionization of the alginate backbone decreases, a number of negatively charged units persists. These units might interact with the positively charged units of P2VP lying within the micellar cores (Figure S2). It seems that this is not the case, which should be attributed to the thick PEO shells that protect the P2VP cores from contact with the grafted alginate. However, some differentiation in T_c,thermo-thickening_ between the composite and the pure ALG-g-HG, i.e., shift at lower temperatures for the composite at pH < 5, below the pK_a_ of P2VP (Table S3), shows that ionic interactions between the charged units of P2VP and alginate should not be excluded.

In a previous paper [18] dealing with the thermo-thickening of ALG-g-P(NIPAM–co–NtBAM) hydrogels, it was shown that the ionic interactions between the negatively charged alginate units with the divalent cations of Ca^2+^ resulted in a strong reinforcement of the network, as revealed by a significant increase of the elasticity below and above the transition temperature of thermo-thickening. Interestingly, the presence of the additional ionic interactions slightly affected the transition temperature, i.e., a few degrees lowering for the highest Ca^2+^ concentration. This means that the ionic interactions act independently and do not affect the hydrophobic ones exerted by the PNIPAM-based side chains, inducing thermo-thickening. The above corroborates our interpretation that the significant shift of the characteristics of the transition zone (Table 1 and Table S3) is due to the interactions between the alginate backbone and the side chains, the nature of which is likely for H-bonding, providing that at low pH, most of the carboxylic units are in their protonated form.

### 3.2. Self-Healing and Injectability

As it is known, these kinds of physically crosslinked hydrogels exhibit a finite linear viscoelastic regime, the extent of which depends on the network structure. Above a certain critical strain (γ_c_), a gel–sol transition occurs due to the mechanical disruption of the physical crosslinks. This γ_c_ was determined at 51%, conducting a strain sweep test at 37 °C on the formulation of pH 4.0 (Figure S4), where a reinforced network was observed.

**Figure 7:**
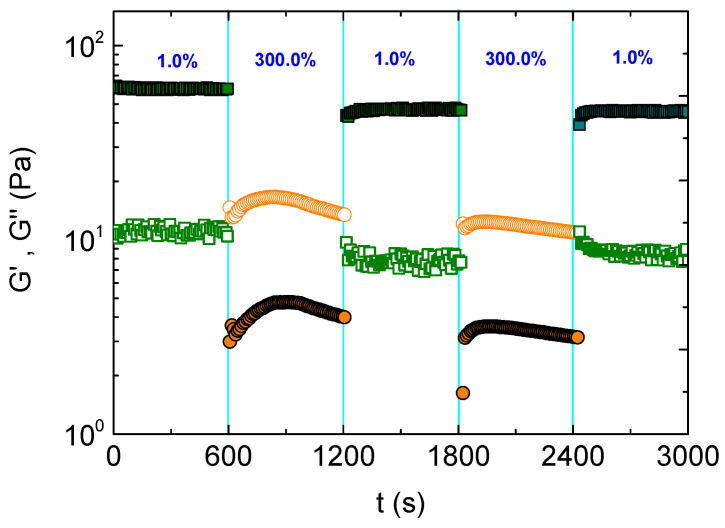
Time dependence of G’ (solid symbols) and G” (open symbols), subjected to consecutive variations of strain amplitude, for the ALG-g-HG/P2VP-*b*-PEO composite hydrogel at pH 4.0 and 37 °C.

To evaluate the self-healing of this hydrogel, successive oscillatory, time sweep experiments were performed by applying stepwise strains below and above γc. Particularly, the hydrogel was subjected to strain amplitudes at 1% and 300% for 600 s at each step, at 37 °C. As seen in Figure 7, the formulation exhibits gel-like behavior (G’ > G”) at 1% strain, which turns instantly to sol (G’ < G”) upon applying 300% strain. The hydrogel was recovered instantly in the third step by switching the strain amplitude again to 1%, within the linear regime. The hydrogel recovery was reproducible, as shown in the fourth and fifth steps. The above results confirmed the self-healing ability of the hydrogel since it is recovered after a sudden network disruption. The self-healing property concerns the application of injectability and 3D printing of the hydrogels. The underpinning physical network is disrupted at high shears, which were applied during these procedures, and it self-heals instantly upon cessation of the shear forces, due to the reformation of the physical network junctions.

**Figure 8:**
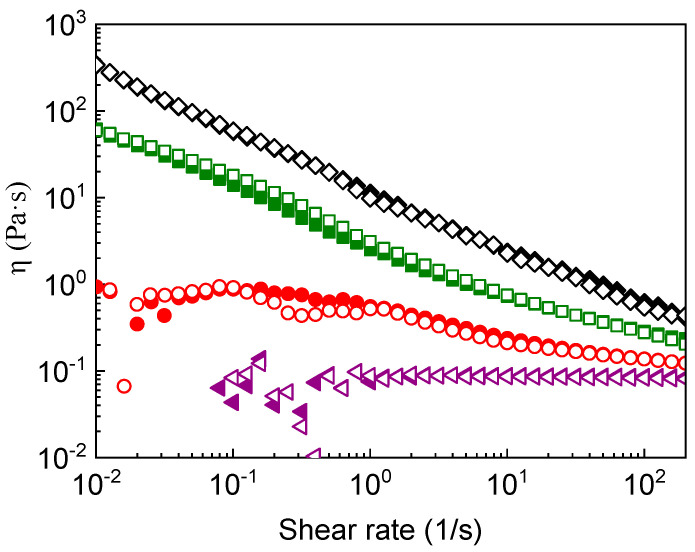
Apparent shear viscosity as a function of shear rate (increasing (open symbols)/decreasing (solid symbols)) obtained at 20 °C for the ALG-g-HG/P2VP-*b*-PEO composite hydrogel at all pHs: pH 3.5 (black symbols); pH 4.0 (green symbols); pH 4.5 (red symbols); pH 5.4 (purple symbols).

Concerning hydrogel performance (e.g., 3D printing), injectability is one of the critical properties of hydrogels as carriers of payloads. This property can be easily afforded when the thermo-responsive hydrogel exhibits a sol–gel transition above room temperature since it flows below the gelation temperature. This is the case for all the formulations for pH > 4.0, while for the system at lower pH (pH 3.5), a gel-like behavior is valid even at room temperature. In the latter case, shear thinning behavior is demanded. Hence, shear flow experiments were conducted to evaluate the response of the hydrogels to shear. Figure 8 displays the apparent shear viscosity (η/Pa·s) as a function of the shear rate (increasing/decreasing) obtained at 20 °C for all pHs. Obviously, the viscosity increased by lowering the pH in any shear rate examined, exhibiting shear thinning except for that at pH 5.4 where a Newtonian behavior was observed. Notably, the shear thinning is perfectly reversible practically without hysteresis since the viscosity values were recovered by decreasing the shear rate. Moreover, the extent of shear thinning augments when the viscosity increases by lowering the pH. For instance, the viscosity drops more than 500-fold by progressively increasing the shear rate from 0.01 to 100 s^−1^ for the formulation of pH 3.5, exhibiting the highest elasticity, while at pH 4.0, the viscosity decrease is about 200 times lower.

**Figure 9:**
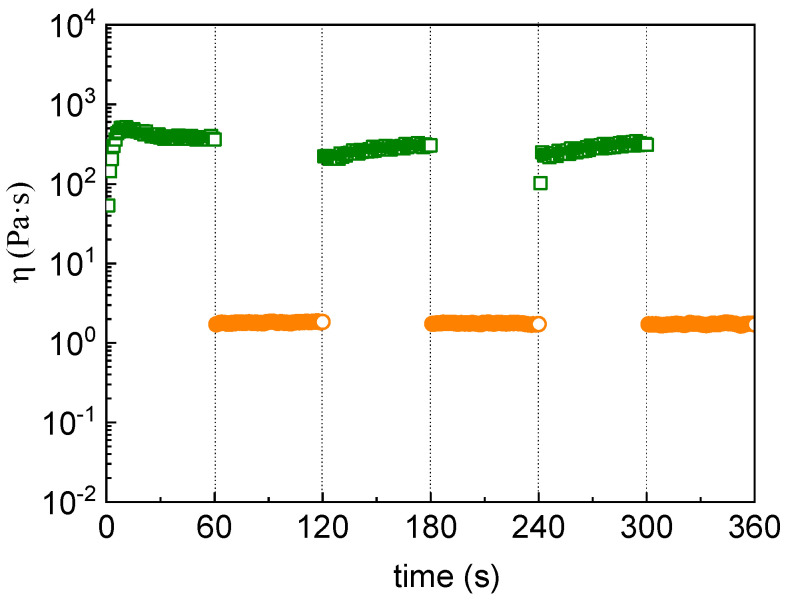
Time dependence of apparent shear viscosity subjected to consecutive variations of shear rates from 0.01 (□) to 20 s^−1^ (o) for the ALG-g-HG/P2VP-*b*-PEO composite hydrogel at pH 3.5 and 20 °C.

Finally, the response of viscosity to shear was evaluated by sudden changes in the applied shear rate as demonstrated in Figure 9. Specifically, the formulation of the highest elasticity (pH 3.5) was subjected to successive stepwise changes of shear rates from 0.01 (approaching situation at rest) to 20 s^−1^ (situation under shear). As observed, the viscosity drops about two orders of magnitude, and it is recovered promptly and reproducibly. Note that the shear rate that is applied during the real injection through a syringe depends on the syringe/needle characteristics and the applied flow rate. For instance, the shear viscosity should be lower than 0.35 Pa·s, when using a 25G needle with a 1 mL/min injection rate, to ensure comfortable injection (see Supplementary Materials) [56]. Provided that the applied shear rate is of the order of thousands s^−1^ (see Supplementary Materials) and that the extrapolated at 10^3^ s^−1^ shear viscosity for the formulation of pH 3.5 (thickest gel) is about 0.16 Pa·s (Figure 8), the present system is considered to be injectable. In conclusion, the results of the experiments demonstrated in Figure 7, Figure 8 and Figure 9 showed that this kind of hydrogel exhibits reversible shear thinning, responding perfectly to sudden changes of shear and self-healing properties, meeting the requirements of 3D printable and injectable hydrogels.

### 3.3. Evaluation as Drug Delivery System

In order to evaluate the effectiveness of the nanocomposite hydrogel as a pH-controlled drug delivery system, the micellar P2VP-*b*-PEO NPs were loaded with the hydrophobic Nile Red, which was encapsulated in their hydrophobic cores, and its release kinetics was conducted at the physiological temperature at two different pH.

It was found that the P2VP-*b*-PEO@Nile Red micelles displayed Nile Red loading efficiency, LE = 1.79% (*w*/*w*), for a theoretical (feed) loading, Lth = 9.98% (*w*/*w*), and encapsulation efficiency, EE = 17.93% (*w*/*w*).

**Figure 10:**
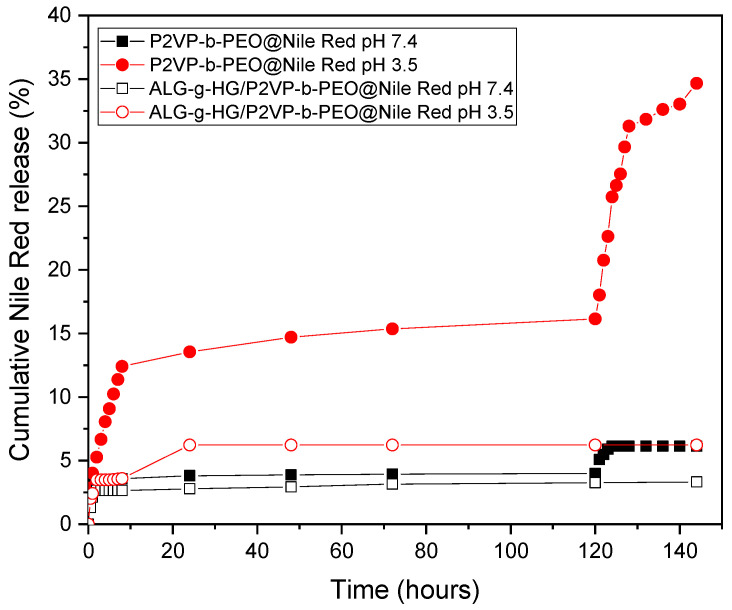
Release of: Nile Red from P2VP*-b-*PEO@Nile Red micelles and ALG-g-HG/P2VP-*b*-PEO@Nile Red nanocomposite hydrogels at pH 3.5 and pH 7.4 buffer solutions and at 37 °C.

The Nile Red-loaded P2VP-*b*-PEO micelles (P2VP-*b*-PEO@Nile Red) and ALG-g-HG//P2VP-*b*-PEO@Nile Red nanocomposite hydrogels were studied to evaluate the Nile Red release. Figure 10 presents the cumulative release of Nile Red versus time for both nanocarrier systems, at 37 °C and at two different buffer solutions of pH 3.5 (similar to the pH of gastric acid in the human stomach) and pH 7.4 (simulates the pH of blood and pH of healthy tissues). From the release study of the P2VP-*b*-PEO@Nile Red micelles, it appears that the release process is highly influenced by the pH of the medium. The micelles in buffer pH 3.5 release a higher amount of Nile Red (16% of Nile Red is released at 120 h) than the micelles in buffer pH 7.4 (4% of Nile Red is released at 120 h). This is due to the protonation of the P2VP moieties of the P2VP-*b*-PEO polymer that makes them less hydrophobic, leading to micelles with a less dense P2VP core. This way, the Nile Red molecules can escape more easily from the hydrophobic P2VP micelle cores.

Table S4 in the Supplementary Material summarizes the particle size distributions of the P2VP-*b*-PEO NPs at three different pH values of the aqueous medium. As observed, when the pH was adjusted to a lower value of pH 3.5, the micellar diameter was substantially decreased to ~3.5 nm. In such an acidic environment, the degree of ionization of P2VP blocks increases by protonation, leading to the disassembly of the micelles into smaller associates or even unimers. These results are in good agreement with those previously reported for copolymers with similar PEO/P2VP(P4VP) hydrophilic/hydrophobic balance [57,58,59]. Thus, one would expect an acceleration of the Nile Red release from the P2VP-*b*-PEO micelles at pH 3.5, where the P2VP blocks are partially charged (Figure S2). However, only 16% of Nile Red was achieved at 120 h, at pH 3.5. This probably could be attributed to the fact that Nile Red is a molecule of high hydrophobicity that could also form π–π stacking interactions with the PVP blocks, making it firmly retained in the polymer micelles.

At pH 7.4, an initial burst release can be observed, which may imply that some Nile Red molecules are weakly bound within the micelle cores and that a fraction of the encapsulated Nile Red is located at the core/corona interface of the micelles and therefore it is more accessible and prone to release. In this pH, high above the pK_a_ of P2VP at pH 5.0, the P2VP moieties are hydrophobic. The low release of 4% of Nile Red in buffer pH 7.4 could be attributed to the highly hydrophobic nature of Nile Red and possible strong, non-covalent interactions of Nile Red with the P2VP hydrophobic micellar cores causing a more firmly Nile Red entrapment in the P2VP-*b*-PEO micelles. These findings are in agreement with the pH-dependent release profiles of various hydrophobic drugs from poly(ethylene glycol)-*b*-poly(2-vinylpyridine) micelles. It should be mentioned that the release kinetics of hydrophobic drugs are remarkably influenced by their chemical structure except for the size and the degree of protonation (controlled by pH) of the P2VP blocks forming the hydrophobic cores [59].

Inspired by the study of Chroni et al. [60], which proved that ultrasound has a significant influence on the release profile of curcumin (CUR) from CUR-loaded nanocarriers, we proceeded to a similar study. Therefore, after the 120 h of the release experiments, the samples were subjected to ultrasonication. It can be clearly seen that for pH 3.5, after 8 h under ultrasound stimulation, a high release of Nile Red occurred (release rate increased from 16% to 31% at 128 h (total experiment hours). After further sonication, for 16 more hours (to reach 144 total experiment hours), Nile Red release increased further to 35%. The increase of Nile Red release from the micelles submerged in the PB buffer of pH 7.4 was marginal, showing a rise of only 2% after 24 h of sonication (from 4% at 120 h, the release of Nile Red reached 6% at 144 h). This release resistance under sonication confirms the strong attractive interactions between Nile Red and the vinyl pyridine moieties of the micellar cores (see also reference [61]), which justifies the observed release kinetics.

When the P2VP-*b*-PEO@Nile Red micelles were loaded in the ALG-g-HG hydrogel, it can be seen, that for both PB buffer solutions at pH 3.5 and pH 7.4, Nile Red release is lower, at around 6% and 3.3%, suggesting a noticeable slowdown of the Nile Red release rate, which is attributed to the high viscosity of the hydrogel medium [50]. Importantly, this effect was more pronounced at pH 3.5. This behavior is related to the pH-induced alteration of the network structure formed by the ALG-g-HG gelator. As already discussed in Figure 2 and Figure 3, the network is reinforced by decreasing pH as reflected in G’ augmentation, due to additional crosslinking induced by H-bonding. Particularly, the complex viscosity η* increased remarkably with the pH reduction at 37 °C, where the release experiments were conducted, as demonstrated in Figure S5. This effect might justify the deceleration of the Nile Red release rate due to the decrease of drug diffusion within the higher viscosity hydrogel medium.

## 4. Conclusions

The pH-dependent properties of LCST-type thermo-responsive alginate-based hydrogels incorporating poly(2-vinyl pyridine)-*b*-polyethylene oxide (P2VP-*b*-PEO) micellar nanoparticles were explored by rheology. Due to the thermo-responsive side chains, the ALG-g-HG copolymer (4 wt%) self-assembles upon heating, exhibiting thermo-thickening behavior above a sol–gel transition T_gel_. Interestingly, the study at a low pH range of just below 5.0 revealed a notable increase in the elasticity of the polymer solution occurring even at low temperatures below T_gel_. This effect transformed the sol–gel to a soft gel–strong gel transition. It was proved that this was not due to the interactions with the protonated P2VP (pH < pK_a_) of the diblock copolymer, provided that this effect was similar to the formulation in the absence of P2VP-*b*-PEO copolymer. Importantly, the onset of thermo-thickening shifted notably to lower temperatures followed by the broadening of the transition zone, implying secondary intermolecular interactions between the protonated repeating units of the alginate backbone with the PNIPAM side chains, likely through H-bonding.

Evaluating the ALG-g-HG/P2VP-*b*-PEO composite hydrogel as a drug delivery system, it was found that the release of a hydrophobic model drug (Nile Red) is affected by the pH of the medium. At pH 3.5, the release of Nile Red was decelerated, due to the additional H-bonding that increases the crosslinking density of the hydrogel network and in turn the viscosity of the hydrogel medium. Moreover, the shear-thinning behavior in combination with the thermo-thickening characteristics of the material, make it a good candidate for potential applications such as a 3D printable composite carrier of payloads in biomedicine.

## Figures and Tables

**Table 1:** Characteristic factors of thermo-responsiveness at various pHs.

ALG-g-HG/P2VP-*b*-PEO					
pH	T_c,thermo-thickening_ (°C)	T_gel_ (°C)	T_f_ (°C)	ΔT = T_f_−T_c, thermo-thickening_ (°C)	G’(T = 50)/G’(T_c, thermo-thickening_)
5.4	32.6	34.1	41.7	9.4	86.2/0.78 = 110.5
4.5	22.3	26.1	40.8	18.5	92.3/0.95 = 97.2
4.0	18.1	20.2	36.6	18.5	244.6/2.8 = 87.3
3.5	19.2	-	38.7	19.5	565.2/160.7 = 3.5

## Data Availability

The raw data supporting the conclusions of this article will be made available by the authors on request.

## Supplementary Materials

The following supporting information can be downloaded at https://www.mdpi.com/article/10.3390/polym16070886/s1, Table S1: Molecular characteristics of the ALG-g-HG heterograft copolymer; Table S2: Molecular characteristics of the P2VP-*b*-PEO copolymer; Table S3: Characteristic factors of thermo-responsiveness at various pHs for the pure ALG-g-HG copolymer; Table S4: The hydrodynamic diameters of P2VP-*b*-PEO polymeric micelles obtained from DLS analyses; Figure S1: Storage (G’) and loss (G”) modulus as a function of temperature of ALG-g-HG solutions (a) 5 wt% and (b) 4 wt%; Figure S2: Zeta potential of aqueous P2VP-*b*-PEO and ALG-g-HG solutions at different pH; Figure S3: Storage (G’) and loss (G”) modulus as a function of temperature of 4 wt% ALG-g-HG hydrogels at (a) pH 4.5 and (b) pH 3.5; Figure S4: Oscillatory strain sweep data at 1 Hz of the 4 wt% ALG-g-HG hydrogel at pH 4 and 37 °C; Figure S5: pH dependence of complex viscosity (1Hz) at 37 °C. Ref. [62] is cited in the Supplementary Materials.
