# Supplementary material for: A Remarkable Impact of pH on the Thermo-Responsive Properties of Alginate-Based Composite Hydrogels Incorporating P2VP-PEO Micellar Nanoparticles
Source: Polymers (Basel). 2024 Mar 24;16(7):886. doi: 10.3390/polym16070886 (PMC11013835; doi:10.3390/polym16070886)
Supplement: Supplementary file 1 [file polymers-16-00886-s001.zip › polymers-2916677-supplementary.pdf]

Supporting Information

A Remarkable Impact of pH on the Thermo-responsive Properties of Alginate-Based Composite Hydrogels Incorporating P2VP-PEO Micellar Nanoparticles

Amalia Iliopoulou[1], Zacharoula Iatridi[1], Constantinos Tsitsilianis[1,*]

[1]Department of Chemical Engineering, University of Patras, 26500 Patras, Greece

Table S1. Molecular characteristics of the ALG-g-HG heterograft copolymer.

**ALG-g-HG heterograft copolymer**	
**Molecular weight, Mw (g/mol)**	262,000
**Average number of P(NIPAM$_{86}$-co-NtBAM$_{14}$) chains per Alginate backbone**	3.5
**Average number of PNIPAM chains per Alginate backbone**	1.8

Table S2. Molecular characteristics of the P2VP-*b*-PEO copolymer.

	**Composition***	**Molecular weight****	
	**(P2VP %moles)**	**Mn (g/mol)**	**Mw (g/mol)**
**P2VP block**	100***	2583	2678
**P2VP-*b*-PEO**	9	13144	14384

*by ^{1}H-NMR; ** by Size Exclusion Chromatography; *** The P2VP block polymer is basically the precursor block on which the ethylene oxide (EO) comonomer is subsequently polymerized to produce the P2VP-*b*-PEO final copolymer

Table S3. Characteristic factors of thermoresponsiveness at various pH for the pure ALG-g-HG copolymer.

4% Alg-g-(NIPAM$_{86}$NtBAM$_{14}$)-g-PNIPAM					
**PH**	**Tc , thermothickening (°C)**	**Tgel (°C)**	**Tf (°C)**	**ΔT=Tf -Tc , thermothickening (°C)**	**G′(T=50)/G′(T= Tc, thermothickening)**
**7.40***	**29.5**	**31.5**	**36.6**	**7.0**	**142/0.4=340**
**5.80**	**32.6**	**34.4**	**41.7**	**9.1**	**66.4/1.1=60.4**
**4.50**	**24.3**	**29.2**	**42.7**	**18.4**	**73.0/2.8=26.1**
**3.50**	**21.2**	**--**	**37.7**	**16.5**	**689.4/213.6=3.2**

*At pH 7.4 the polymer concentration was 5 wt%.

Table S4. The hydrodynamic diameters of P2VP-*b*-PEO polymeric micelles obtained from the number- and volume- weighted distributions of the DLS analyses. Values are reported as the mean value of the peak size.

Particle Size Distributions		
	**Diameter, nm**	
**pH**	**Number-weighted**	**Volume-weighted**
**7.4**	20.03	22.38
**5.4**	28.92	33.73
**3.5**	3.21	3.50

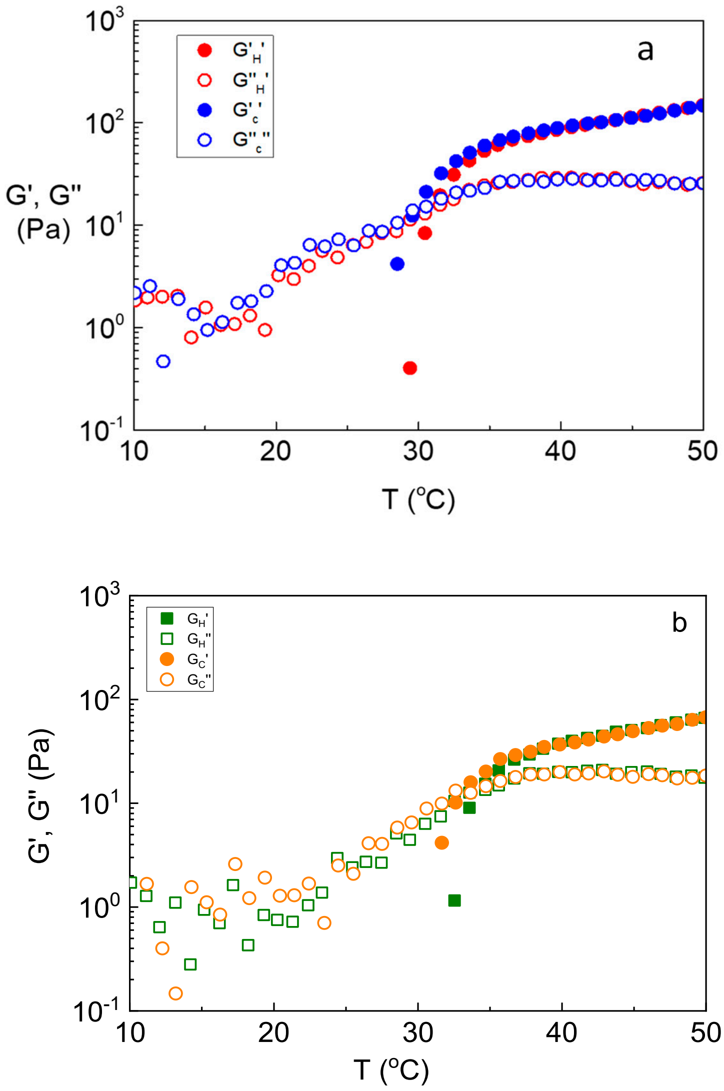

Figure S1. Storage (G') and loss (G") modulus as a function of temperature of ALG-g-HG solutions (a) 5 wt% and (b) 4 wt%.

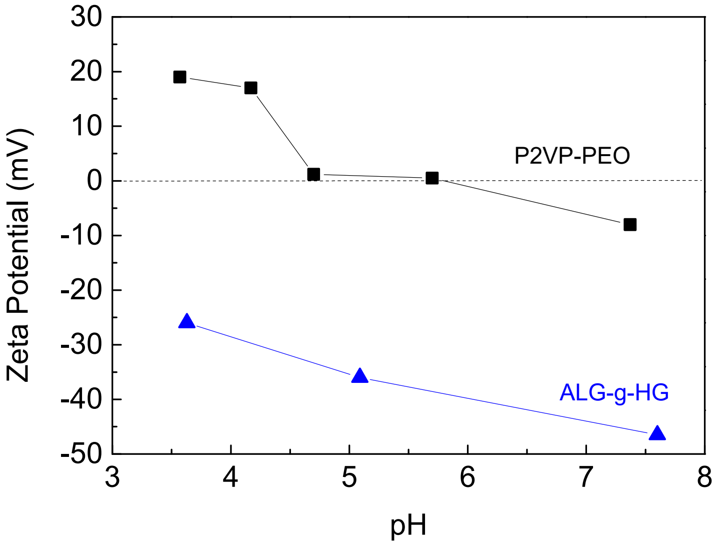

Figure S2. Zeta potential of aqueous P2VP-*b*-PEO (■) and ALG-g-HG (▲) solutions at different pH.

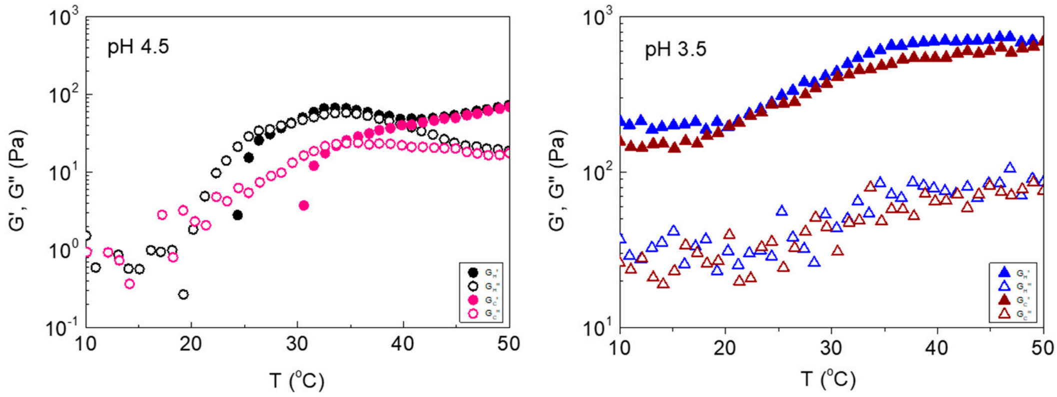

Figure S3. Storage (G′) and loss (G″) modulus as a function of temperature of 4 wt% ALG-g-HG hydrogels at (**a**) pH 4.5 and (**b**) pH 3.5.

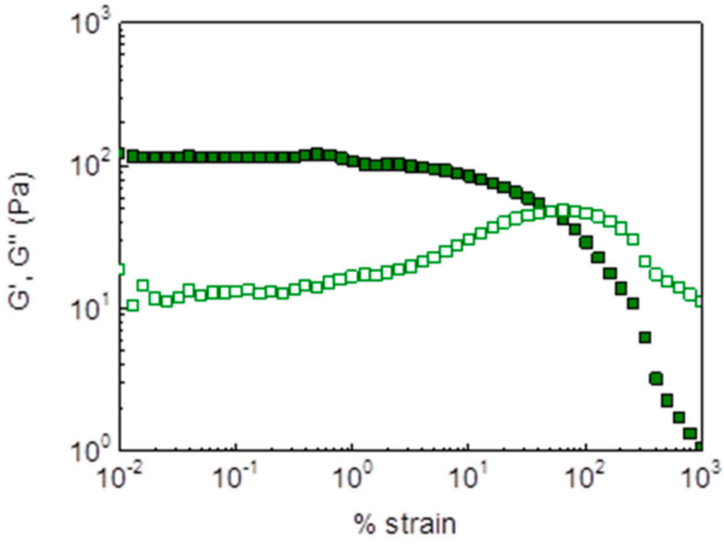

Figure S4. Oscillatory strain sweep data at 1 Hz of the 4 wt% ALG-g-HG hydrogel at pH 4 and 37 °C.

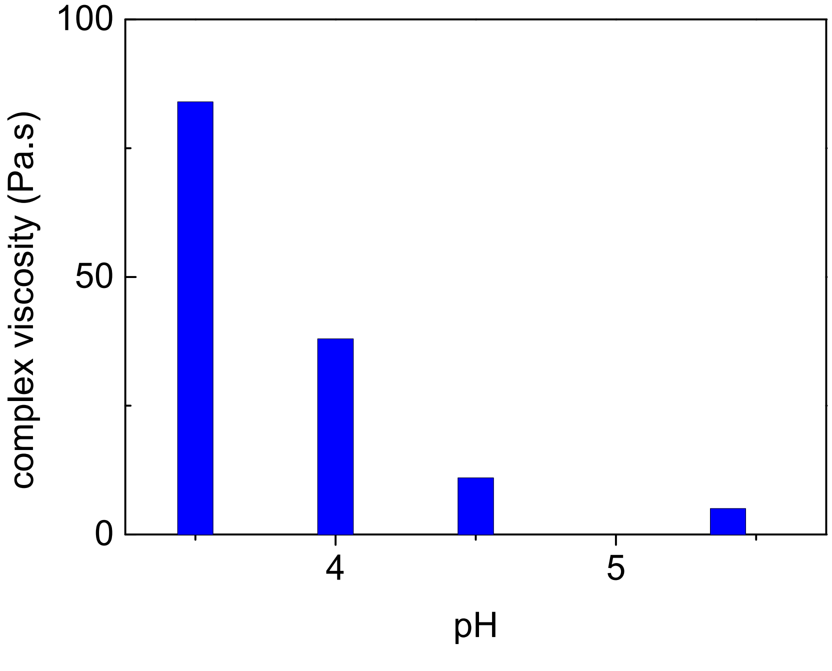

Figure S5. pH dependence of complex viscosity (1Hz) at 37 °C. The data were extracted from the experiments of Figure 2 (heating ramp).

Injectability

As reported the limit of the injection force F for a comfortable injection is 12 N [1]. F is given by the equation 1, where η is the shear viscosity and Q_v the flow rate of a liquid injected through a syringe of radius R_s, needle of radius R_n, and length L, with F_f the friction force of the piston in the syringe [2].

$$F = \frac{8\eta L Q_v R_s^2}{R_n^4} + F_f \qquad (1)$$

For a 25G syringe: R_s=2.4 mm, R_n=0.13 mm, L=12.7 mm. Considering that the friction force is negligible (F_f=0) and applying Q_v=1mL/min, equation 1 can be written as F=Kη where K=34.5 $m^2\ s^{-1}$

For a comfortable injection, F should be lower than 12 N [1]. Therefore, the shear viscosity should be lower than η=F/K=12 N/34.5 $m^2\ s^{-1}$=0.35 Pa.s.

Moreover, the shear rate $\dot{\gamma}$ applied during injection can be calculated by the equation (2).

$$\dot{\gamma} = \frac{4Q_v}{\pi R_n^3} \qquad (2)$$

For a 25G syringe (R_n=0.13 mm) and Q_v=1mL/min $\dot{\gamma}$=9.65 x $10^3\ s^{-1}$.

References

[1] T. E. Robinson, E. A. B. Hughes, A. Bose, E. A. Cornish, J. Y. Teo, N. M. Eisenstein, L. M. Grover, S. C. Cox Filling the Gap: A Correlation between Objective and Subjective Measures of Injectability. *Adv. Healthcare Mater.* **2020**, *9*, 1901521 DOI: 10.1002/adhm.201901521

[2] A. Allmendinger, S. Fischer, J Huwyler, H.-C. Mahler, E. Schwarb, I. E. Zarraga, R. Mueller, Rheological characterization and injection forces of concentrated protein formulations: An alternative predictive model for non-Newtonian solutions. *European Journal of Pharmaceutics and Biopharmaceutics* **2014**, *87*, 318–328, doi.org/10.1016/j.ejpb.2014.01.009
